# A model for the co-evolution of dynamic social networks and infectious disease dynamics

**DOI:** 10.1186/s40649-021-00098-9

**Published:** 2021-10-07

**Authors:** Hendrik Nunner, Vincent Buskens, Mirjam Kretzschmar

**Affiliations:** 1 Department of Sociology/ICS, Utrecht University, Padualaan 14, 3584 CH Utrecht, The Netherlands; 2 Centre for Complex Systems Studies (CCSS), Utrecht University, Leuvenlaan 4, 3584 CE Utrecht, The Netherlands; 3 Julius Centre for Health Sciences and Primary Care, University Medical Centre Utrecht, Universiteitsweg 100, 3584 CG Utrecht, The Netherlands; 4 Centre for Infectious Disease Control, National Institute for Public Health and the Environment (RIVM), P.O. Box 1, 3720 BA Bilthoven, The Netherlands

**Keywords:** Network formation, Complex networks, Network dynamics, Epidemics, Infectious diseases, Health behavior, Risk perception, Agent-based simulation, Primary 91D30, Secondary 05C82

## Abstract

Recent research shows an increasing interest in the interplay of social networks and infectious diseases. Many studies either neglect explicit changes in health behavior or consider networks to be static, despite empirical evidence that people seek to distance themselves from diseases in social networks. We propose an adaptable steppingstone model that integrates theories of social network formation from sociology, risk perception from health psychology, and infectious diseases from epidemiology. We argue that networking behavior in the context of infectious diseases can be described as a trade-off between the benefits, efforts, and potential harm a connection creates. Agent-based simulations of a specific model case show that: (i) high (perceived) health risks create strong social distancing, thus resulting in low epidemic sizes; (ii) small changes in health behavior can be decisive for whether the outbreak of a disease turns into an epidemic or not; (iii) high benefits for social connections create more ties per agent, providing large numbers of potential transmission routes and opportunities for the disease to travel faster, and (iv) higher costs of maintaining ties with infected others reduce final size of epidemics only when benefits of indirect ties are relatively low. These findings suggest a complex interplay between social network, health behavior, and infectious disease dynamics. Furthermore, they contribute to solving the issue that neglect of explicit health behavior in models of disease spread may create mismatches between observed transmissibility and epidemic sizes of model predictions.

## Introduction

In an increasingly globalized world, we face risks of outbreaks of infectious diseases, such as AIDS, Ebola, COVID-19, or new strains of influenza. Human behavior is known to have major influence on infectious disease dynamics [1]. Examples are how we interact with one another when we are sick, whether we break social ties over the course of infections, what we believe to know about health risks, and how we derive actions from this information.

Past studies show an increasing interest in this interplay of health behavior and infectious diseases in both sociological and epidemiological scholarship [2–6]. Mao et al. [7], for example, emphasize that neglect of health behavior may create mismatches between observed transmissibility of diseases and epidemic sizes of model predictions. Based on a 2-layer network (contact, information) they predict lower epidemic sizes when more individuals adopt preventive behaviors disseminated through communication networks. Further integration of health behavior, the authors argue, is necessary and requires an interdisciplinary approach combining social sciences, health psychology and epidemiology.

From studies in health psychology we know, for example, that perceived risks of infections may cause people to avoid social gatherings during times of increased risks of infection [8, 9]. Despite the widely recognized importance of incorporating dynamical aspects in epidemiological contexts [10, 11], many studies on infectious diseases consider social networks to be static, implying that health behavior does not affect social behavior. Static relations, however, do not describe the dynamic nature of our social networks [12, 13], while disease dynamics is known to be sensitive to topology changes in adaptive networks [14].

Leung et al. [15] recently addressed the problem of lacking social network dynamics. Using a formal model of disease spread, they showed that social distancing implemented by stochastic rewiring of network ties, rather than dropping edges, and thus keeping average degree constant, has an immediate effect on the epidemic size. High rewiring rates of susceptible agents when connected to infected peers result in a lower epidemic size than in static networks. Moderate rewiring rates, in contrast, result in larger epidemic sizes. The authors propose, however, that individual decision-making is a non-stochastic, but highly complex health behavioral process waiting to be formally integrated into epidemiological social network models. We posit that a more realistic description of epidemics is possible by integrating social network dynamics based on health behavior into epidemiological models. This paper identifies the necessary theories and provides a general model to fill this gap.

Considering health behavior, Funk et al. [16] argue that people need to become aware of disease-related information (e.g., personal observations, public information campaigns) to change their behavior. Further, two systematic reviews on airborne diseases [17, 18] conclude that subjective risk perception with regard to (i) the perceived probability of getting infected and (ii) the perceived severity of a disease is the main reason for health-related behaviors in the context of infectious diseases. Thus, a person perceiving risk to be high may prefer to avoid social contacts during a wave of influenza, while a person perceiving risk to be low may not alter social behavior at all [see also, 9, 11, 19]. The model by Poletti et al. [20] also suggests effects on epidemic impact depending on perceived risks and the corresponding behavioral reaction (lowering probability of infection), even for small reductions in contact numbers. Finally, Bish et al. [17] argue that the number and severity of symptoms are significant factors for the duration and degree of separation from social contacts.

The elaborations above demonstrate a complex co-evolution of infectious disease dynamics and social network dynamics mediated by risk perceptions of actors. It remains, however, unclear how this co-evolution comes about. We therefore ask: *How does health behavior shape the co-evolution of epidemics and dynamic social networks?* Despite the enormous increase in the number of studies of disease spread in social networks since the beginning of the COVID-19 pandemic [e.g., 21–34], we are not aware of any study explicitly considering a choice-based model including health behavior and interdependent dynamics of social networks and infectious disease spread. Modeling such network-related health behavior allows to compare the effects of different control measures against epidemic outbreaks, a crucial factor for policy-making [1, 35]. Furthermore, our work adds to the rich literature on co-evolution between social networks and different types of behavior [36, 37].

To answer the question, we provide a general model for the co-evolution of dynamic social networks and infectious diseases mediated by risk perceptions of actors. The model follows an interdisciplinary approach and combines theories of social network formation from sociology and economics, infectious diseases from epidemiology, and individual health behavior from health psychology. Furthermore, rather than using purely stochastic rewiring processes or artificially lowering transmissibility [see 3, 7, 15], we address two crucial shortcomings of contemporary models to couple the dynamics of social networks and disease behavior based on a more explicit theoretical mechanism: (i) the integration of dynamic network structure in consideration of (ii) behavioral changes due to infection risks [38].

We first identify the minimal requirements for such a model with solid footing in the literature (“Theory” section), including theory from epidemiological scholarship [39] and theory on how people form social ties [40–42]. In addition, we use psychological theory to describe health behavior and how people translate the exposure to health risks into action. We integrate these theories to propose a general model describing the co-evolutionary processes of dynamic social networks and infectious diseases (“The networking during infectious diseases model (NIDM)” section). A specific model case (“A specific model case” section) implemented as agent-based simulation (“Simulation” section), serves to illustrate how the general model can be tied to specific types of social networks (here: 10 to 50 individuals, such as groups of friends, school classes, extended family, small companies, or teams within larger companies), and how different social, psychological, and disease-related conditions affect the course of epidemics within these networks (“Results and discussion” section).^1^ We conclude with the implications of our study and opportunities for further research “Conclusion and implications” section.

## Theory

### Infectious diseases

Infectious diseases differ in many aspects, such as transmission routes (e.g., airborne, sexual contact, animal vectors, food contamination), the symptoms they cause (e.g., fever, coughing, fatigue, diarrhea, muscle aches), the body parts they affect (lungs, skin, inner organs), virulence, the cause of infection (bacteria, viruses, fungi, parasites), the course and duration of the disease, and many more. However, despite the numerous differences between specific infectious diseases, there are also commonalities that apply to all. The infection rate, or probability of an infection per contact, is central for the spread of any infectious disease and thus an important factor whether the invasion of an infection may turn into an epidemic [16]. Furthermore, the stages individuals go through during an infection are similar. This progress typically starts with an individual being susceptible to a disease. After being infected with a pathogen, the susceptible individual becomes infectious and possibly symptomatic. Finally, the individual either recovers or dies from the infection. Recovered individuals may either be immune or once again become susceptible to the disease.

This general course of infectious diseases can be translated into the well-known compartmental *SIR model* [39], which divides a population into three different compartments according to disease states (Susceptible, Infected, and Recovered). Depending on transmission and recovery rates, ordinary differential equations allow predicting different properties of infectious disease dynamics, such as the total number of infected, peaks and duration of epidemics, and effects of vaccines. Models are designed depending on, inter alia, which states of the infection are considered or whether recovered individuals may die, become immune, or become susceptible again.

Despite the undisputed importance of compartmental models in epidemiology, Wang et al. [38] describe the lack of theoretical foundation in behavioral responses and social mixing as a pitfall. Especially the assumption that individuals have the same probability to get in contact with others is delusive, as people get more likely in contact with others who are close to them (spatially, emotionally, characteristically) [43]. More recent studies addressed this issue by dividing populations into spatially distinct groups, so-called *meta-populations* [2, 44], or using network approaches to study the effect of network topology, interpersonal relationships, informational exchange, or protective behavior on disease spread [7, 10, 14, 15, 45–47]. While static networks provide good approximations when networks change at a much slower pace than diseases spread, an increasing number of studies are concerned with network dynamics and disease dynamics occurring on similar timescales [14]. Model studies on adaptive networks allow unique insights on the non-trivial feedback loop between infectious disease spread and spontaneous changes in human behavior. Game-theoretic approaches study, for example, the conditions that make social distancing a beneficial response to infection risks [5, 48]. Studies to model temporary interruption of contacts between infected actors [6, 49, 50] show that pausing relationships during epidemics increase the epidemic threshold. The underlying network structure in these studies, however, remains static. In a study to model epidemic dynamics in adaptive networks, [4] showed that disease spread is inhibited when susceptible actors choose to distance themselves from infected others. Furthermore, they showed that depending on rewiring rate and initial state of the system, healthy and endemic states can coexist.

While all these approaches impressively show that network dynamics may alter the course of epidemics, they neglect modeling health behavior explicitly.^2^ We therefore seek to identify the relevant theories to describe how people form their networks and how this behavior differs when being at risk of getting infected with a disease.

### Social network formation

Depending on the context, different positions within a network may create structural advantages, which in turn create social or economic benefits [51–54]. As a result, individuals consciously change their networks to their personal advantage [55, 56].

Benefits in our general model are considered personal well-being, a combination of social and physical well-being, as suggested by *Social Production Function* theory [*SPF* theory; 57]. Social well-being can be satisfied through affection or behavioral confirmation by direct personal contacts. This creates an incentive to establish connections to other persons. Maintaining ties, however, comes at the cost of time and effort [41]. It is therefore not necessarily rational for an actor to just randomly connect to everyone else, as some ties might create higher value than others while others have higher costs for maintenance.

According to SPF theory, a major factor of physical well-being is the absence of physical harm. Consequently, the presence of an infectious disease within a social network creates a potential harm to each individual and thus creates an incentive not to form or even to break existing ties with (infectious) others. As a result, networking behavior becomes a trade-off (utility $U_{i}$) of an individual (*i*) between the social well-being his/her contacts create (benefit $B_{i}$), the costs to maintain the ties ($C_{i}$), and the physical harm the connection may cause due to an infectious disease ($D_{i}$):

1 $$\begin{aligned} U_{i} = B_{i} - C_{i} - D_{i}. \end{aligned}$$

The first two components of the equation describe the net social utility of contacts disregarding the potential harm of infections. [41] provides a framework to model this. These *Strategic Network Formation Models* [41, ch. 6] formalize how and why individuals form connections based on the costs and benefits of ties. Analyses of the resulting network and utility structures enable to explain the psychological and societal constraints under which certain network properties come about.

It has to be noted that appropriate models of utility structures depend on the type of disease and its mode of transmission (HIV: sexual contacts, measles: classrooms). Further, potential costs of infections affect expected utility. Actors, however, may apply behavioral changes regardless of the exact shape of the utility function to avoid infections as we argue below.

### Health behavior and risk perceptions

Health behaviors affect many everyday decisions including nutritional issues, personal and sexual encounters, or substance use [11, 58, 59]. Regarding infectious diseases, Bish et al. [17] classify health behaviors into three categories: (i) preventive behaviors (e.g., hand washing, mask wearing, vaccinations); (ii) avoidant behaviors (e.g., work absence during a wave of influenza), and (iii) management of disease behaviors (e.g., consulting medical experts). All these behaviors lower or even eliminate the risks of getting infected. Here, we focus on avoidant behaviors, as we expect it to have the greatest effect on social network structures. For social networks this translates to *avoiding potentially infectious social connections*. Such social distancing can be achieved through either choosing not to form a tie to, or breaking an existing tie from, an infectious other. Empirical studies support that social distancing successfully reduces infection risks through link removal [8], voluntary quarantine [60], or avoidance of public places [9]. Ref. [61] show further that social distancing at the workplace reduces epidemic size. But how do people choose to avoid others when being at risk of infections?

In their systematic review of 30 articles on SARS and avian influenza, [18] describe that independent of how different studies conceptualize risk (i.e., subjective expected utility vs. psychometric), the main commonality among all studies is that risk perception is the driving factor for health-related decisions. Further, risk perception is affected mainly by the perceived susceptibility to and the perceived severity of a disease [17, 18]. A systemic review by [62] emphasizes the subjective nature of perceived susceptibility: people typically make inaccurate predictions about health risks and thus actual and perceived risks of getting infected may differ greatly. While the findings for severity are not as consistent, there is also abundant evidence that the perceived severity of a disease (e.g., expected harm for health, expected fatality) has a major effect on risk assessment as well [18, 62].

A behavioral model that describes how individuals make health-related decisions based on the aforementioned considerations is the ego-centered *Health Belief Model* [in short: *HBM*; 63]. Similar to the HBM, our model integrates the empirically documented concepts *perceived susceptibility* and *perceived severity* of a disease to capture health behavior driven by risk perception.

## The networking during infectious diseases model (NIDM)

Based on the foregoing theoretical considerations a formal representation of networking behavior and infectious diseases needs to satisfy two requirements: first, social distancing is the result of a deliberation process that weighs the net benefits for keeping a connection to an infectious peer and the harm of a disease that potentially results from the same connection. Second, objective measurements for the harm of a disease (susceptibility, severity) need to be modifiable to satisfy the subjective nature of risk perceptions.

The integration of these requirements is expressed by the *Networking during Infectious Diseases Model* (*NIDM*). The NIDM assumes an actor (*i*) to optimize the utility function in Eq. 2 (*U*), an elaborate version of Eq. 1, combining the benefit of social connections ($B_{i}$), the costs to maintain ties ($C_{i}$), and the potential physical harm of infectious contacts ($D_{i}$):

2 $$\begin{aligned} U_{i}({\varvec{G}},{\varvec{d}},{\varvec{R}}) = B_{i}({\varvec{G}},{\varvec{d}}) - C_{i}({\varvec{G}},{\varvec{d}}) - D_{i}({\varvec{G}},{\varvec{d}},{\varvec{R}}). \end{aligned}$$

Utility depends on the network structure (${\varvec{G}}$), the disease state of all actors (${\varvec{d}}$), and their risk perceptions (${\varvec{R}}$). We assume individuals to act boundedly rational using the information available through their environment. That is, individuals act strategically in order to maximize their myopic personal benefits.

In the following, we present a specific case of the NIDM to illustrate how it can be used to study the co-evolution of social networks and infectious diseases for a specific type of social network.

## A specific model case

In a first step to create a specific model from the NIDM, we define a baseline utility function for network formation. This utility function describes the social context and thus determines the properties of the social network to be studied. We choose the truncated version of the *Connections Model* [*CM*; 41, 64]. By choosing a well-known model for an initial investigation, we hope to facilitate understanding of the results. The CM defines utility (*U*) of an actor (*i*) as the combination of benefits $\alpha$ of connections at distance 1 (direct connections), benefits $\beta$ of of connections at distance 2 (indirect connections), and the costs to maintain direct connections *c*:

3 $$\begin{aligned} U_{i} ({\varvec{G}}) = \alpha \cdot n_{i} + \beta \cdot m_{i} - c \cdot n_{i}, \end{aligned}$$

where $n_{i}$ is the number of direct and $m_{i}$ is the number of indirect connections. We use the truncated version implying that only benefits of connections at distances 1 and 2 are considered rather than also providing benefits for longer distances. The model represents that people do not only benefit from direct contacts by receiving help, support, information, etc., but that they can also obtain benefits indirectly from others connected to direct contacts.

Note that the CM is a model typically describing social contexts of limited group size, such as groups of friends, school classes, extended family, small companies, or teams within larger companies. Furthermore, while the CM is an ad hoc choice, it contains characteristics relevant for infectious diseases. Consider a sick person, who receives care by direct friends (doing groceries, taking over chores). In addition, friends of friends may be beneficial, for example by enabling the friend to help (taking over his/her chores) or in the form of practical matters (borrowing an inhaler, providing information on care).^3^ Additionally, the model provides an interesting example for the co-evolution of social networks and infectious diseases as networking behavior depends on benefits from direct and indirect connections, while diseases are transmitted only between actors in direct connection.

We consider connections to be unweighted, undirected, and non-reflexive; presented by the adjacency matrix ${\varvec{G}} = g_{ij}$, with $g_{ij} \in \{0,1\}$, $g_{ii} = 0$, and $g_{ij} = g_{ji} = 1$ if a tie between actors *i* and *j* exists. The *degree* is the number of ties at distance 1 of an actor:

4 $$\begin{aligned} n_{i} = \sum _{j} g_{ij}. \end{aligned}$$

The *distance 2 degree* is defined by the sum of all indirect connections of an actor ($t_{ij}$):

5 $$\begin{aligned} m_{i}= & {} \sum _{j} t_{ij}, {\text{with}} \end{aligned}$$

6 $$\begin{aligned} t_{ij}= & {} {\left\{ \begin{array}{ll} 1, \quad \text {if} \quad ({\varvec{G}}^2)_{ij} > 1 \quad \text {and} \quad g_{ij} = 0 \quad \text {and} \quad i \ne j \\ 0, \quad \text {otherwise}.\end{array}\right. } \end{aligned}$$

We extend the CM by considering a generic infectious disease and health behavior driven by risk perception. The utility in the resulting *Connections during Infectious Diseases Model* (*CIDM*) is therefore only affected when a disease is introduced into the network. We define the vector of disease states for all actors:

7 $$\begin{aligned} \begin{aligned} {\varvec{d}} \in \{S,I,R\}^{N}, \end{aligned} \end{aligned}$$

with $d_{i} = S$ if *i* is susceptible, $d_{i} = I$ if *i* is infected, and $d_{i} = R$ if *i* is recovered.

Consequently, the ties of an actor ($g_{ij}$) can be categorized by disease state. For distance 1, we define:

8 $$\begin{aligned} n_{i_{X}} = \sum _{j,~d_{j} = X} g_{ij}, \quad \text {where} \quad X = S, I, \text { or } R, \end{aligned}$$

as the number of actors at distance 1 with disease state *X*, while for distance 2 that is:

9 $$\begin{aligned} m_{i_{X}} = \sum _{j,~d_{j} = X} t_{ij}, \quad \text {where} \quad X = S, I, \text { or } R. \end{aligned}$$

In addition to the network structure (${\varvec{G}}$), social benefits ($B_{i}$) of actor *i* depend now on the disease state of connected peers (${\varvec{d}}$) :

10 $$\begin{aligned} B_{i}({\varvec{G}},{\varvec{d}}) = \alpha \cdot (n_{i_{S}} + \kappa \cdot n_{i_{I}} + n_{i_{R}}) + \beta \cdot (m_{i_{S}} + \lambda \cdot m_{i_{I}} + m_{i_{R}}), \end{aligned}$$

where $0 \le \kappa \le 1$ is a discount factor for the value of infected direct ($n_{i_{I}}$) and $0 \le \lambda \le 1$ is a discount factor for the value of infected indirect connections ($m_{i_{I}}$). Thus, Eq. 10 captures that infected connections may not be able to provide support as usual. A similar approach is used for the costs of actor *i* to maintain social relations:

11 $$\begin{aligned} C_{i}({\varvec{G}},{\varvec{d}}) = c \cdot (n_{i_{S}} + \mu \cdot n_{i_{I}} + n_{i_{R}}), \end{aligned}$$

where $\mu \ge 1$ is a cost increase for infected direct connections ($n_{i_{I}}$). Therefore, Eq. 11 suggests that maintaining infected connections may result in higher efforts, for example due to nursing care.

(Potential) harm of infections for actor *i* ($D_{i}$) is the product of probability to get infected ($p_{i}$) and severity of the infection ($s_{i}$). $D_{i}$ depends on network structure (${\varvec{G}}$), the disease state of all actors (${\varvec{d}}$), and risk perceptions (${\varvec{R}}$):

12 $$\begin{aligned} D_{i}({\varvec{G}}, {\varvec{d}}, {\varvec{R}}) = p_{i}({\varvec{G}}, {\varvec{d}}, {\varvec{R}}) \cdot s_{i}({\varvec{d}},{\varvec{R}}). \end{aligned}$$

In order to model the subjective nature of risk perceptions, we distinguish between two modifiers:

13 $$\begin{aligned} {\varvec{R}}: {\varvec{r}}_{\pi } \in \mathbb {R},~{\varvec{r}}_{\sigma } \in \mathbb {R}, \end{aligned}$$

where $r_{\pi }$ modifies the actual probability to get infected (see Eq. 14) and $r_{\sigma }$ modifies actual severity of the infection (see Eq. 16).

The probability to get infected is divided into three different cases, depending on an actor’s own disease state:

14 $$\begin{aligned} p_{i}({\varvec{G}}, {\varvec{d}}, {\varvec{R}}) = {\left\{ \begin{array}{ll} \pi _{i}({\varvec{G}},{\varvec{d}})^{2-r_{\pi }}, \quad \text {if} \quad d_{i} = S \\ 1, \quad \text {if} \quad d_{i} = I \\ 0, \quad \text {if} \quad d_{i} = R. \end{array}\right. } \end{aligned}$$

In case an actor is in the recovered state ($d_{i} = R$) the probability to get infected is 0 (immune). In case the actor is infected ($d_{i} = I$), the infection is present ($p_{i} = 1$). If an actor is susceptible ($d_{i} = S$), the probability to get infected depends on two factors. First, the actual probability to get infected:

15 $$\begin{aligned} \pi _{i}({\varvec{G}},{\varvec{d}}) = 1 - (1 - \gamma )^{n_{i_{I}}}, \end{aligned}$$

where $\gamma$ is the objective probability to get infected per single contact. Second, the risk perception factor for the probability to get infected ($0 \le r_{\pi } \le 2$). The power function accounts for the uncertainty of actors with regard to their own susceptibility. Note that we use $2-r_{\pi }$ as the exponent so that the interpretation of $r_{\pi }$ is such that if $r_{\pi }$ increases, actors subjectively estimate the risk of infection to be higher. Thus, $r_{\pi } = 1$ implies an accurate estimate, while $r_{\pi } < 1$ and $r_{\pi } > 1$ represent an underestimation and an overestimation of personal susceptibility, respectively.

Finally, disease severity ($s_{i}$) describes how strongly an actor is affected by the symptoms of the disease. Perceived severity of the disease is again dependent on an actor’s disease state (${\varvec{d}}$) and risk perceptions (${\varvec{R}}$):

16 $$\begin{aligned} s_{i}({\varvec{d}}, {\varvec{R}}) = {\left\{ \begin{array}{ll} \sigma ^{r_{\sigma }}, \quad \text {if} \quad d_{i} = S \\ \sigma , \quad \text {if} \quad d_{i} = I \\ 0, \quad \text {if} \quad d_{i} = R. \end{array}\right. } \end{aligned}$$

An actor in the recovered state ($d_{i} = R$) is immune and thus cannot be affected by the disease ($s_{i} = 0$). Infected actors ($d_{i} = I$) experience the objective severity of the disease ($s_{i} = \sigma$, with $\sigma > 1$). For susceptible actors ($d_{i} = S$), however, the risk perception factor ($0 \le r_{\sigma } \le 2$) transforms actual severity into subjectively perceived risks of a disease.

## Simulation

In the following, we illustrate the framework for agent-based CIDM simulations. First, we describe the simulation procedure. Parameter settings used to study model behavior are explained thereafter.

### Simulation procedure

A single simulation consists of two stages, each composed of a number of time steps (iterations): (i) network initialization (150 time steps) and (ii) epidemic (200 time steps). The number of time steps is fixed to standardize subsequent analyses. It is also sufficiently large to always attain *pairwise stable* networks [64] at the end of both stages, and disease-free networks at the end of the epidemic stage. Pairwise stability means that no agent either benefits from breaking an existing tie unilaterally and no pair of actors from creating a non-existing tie. Pairwise stability is tested at the end of each time step by checking for all possible pairs of agents whether adding a non-existing tie or removing an existing tie increases utility.

In the first time steps of the epidemic stage a single randomly selected agent is infected with a communicable disease. By introducing the disease into a pairwise stable network we ensure that changes in network structure are solely based on health behavioral reactions of the agents.

A single time step consists of two distinct, consecutive, and interdependent processes: (i) disease dynamics followed by (ii) social network dynamics. Disease dynamics define the transmission of infections between agents:

- Repeat until all agents have been processed:
  - Randomly select an unprocessed agent *i.*
  - If *i* is infected, compute whether agent recovers (passed time steps since infection $\ge \tau$).
  - If *i* is susceptible, compute whether *i* gets infected from infected direct connections (Eq. 15).

Social network dynamics define the formation and termination of ties between agents:

- Repeat until all agents have been processed:
  - Randomly select an unprocessed agent *i.*
  - *i* randomly retrieves a proportion $\phi$ of all other agents *j* in the network ( $\phi \cdot (N - 1)$).
  - Repeat until all retrieved agents have been processed:
    - Randomly select (another) retrieved co-agent *j.*
    - If *i* is connected to *j*:
      - Terminate tie *ij*, if the utility for *i* without *ij* is larger than the current utility with *ij*.
    - If *i* is not connected to *j*:
      - Form tie *ij*, if utility for both *i* and *j* is larger with *ij* than current utility without *ij*.

### Parameter settings

Table 1 shows an overview of admissible and used CIDM parameters. These parameters fall into two categories: (i) ego-centered utility parameters and (ii) network parameters. We decided on a minimal but expressive selection of parameter variations that allows to investigate the effects of each model component on overall behavior. That is, we varied only one parameter per term in the utility function (i.e., benefit of indirect ties for social benefits, cost increase for infected direct ties for social maintenance costs, disease severity as property of diseases and risk perception as property of agents for potential harm of infections), and selected settings that allow strong variations in dynamics. Further, we used a limited number of fixed values rather than randomized values for each simulation run. This allows to disentangle model behavior with regard to parameter settings and stochastic processes of the simulations, and to exercise more control over the parameter combinations we believe to be of interest.

**Table 1:** Overview of CIDM parameters

Parameter	Admissible	Used
I. Utilities		
I.I. Social benefits ($B_{i}$)		
Benefit of direct ties	$\alpha \in \mathbb {R}$	$\alpha = 10$
Discount of infected direct ties	$0 \le \kappa \le 1$	$\kappa = 1.0$
Benefit of indirect ties	$\beta \in \mathbb {R}$	$\beta \in \{2, 8\}$
Discount of infected indirect ties	$0 \le \lambda \le 1$	$\lambda = 1.0$
I.II. Social maintenance costs ($C_{i}$)		
Costs to maintain direct ties	$c \in \mathbb {R}$	$c = 9$
Cost increase for infected direct ties	$\mu \ge 1$	$\mu \in \{1.0, 1.5\}$
I.III. Potential harm of infections ($D_{i}$)		
Disease severity	$\sigma > 1$	$\sigma \in \{2, 10, 50\}$
Probability of getting infected per contact	$0 \le \gamma \le 1$	$\gamma = 0.1$
Risk perception (disease severity)	$0 \le r_{\sigma } \le 2$	$r_{\sigma } = r_{\pi } = r \in \{0.5, 1.0, 1.5\}$
Risk perception (probability of infection)	$0 \le r_{\pi } \le 2$	
Recovery time	$\tau > 0$	$\tau = 10$
II. Network		
Network size	$N > 1$	$N \in \{10, 15, 20, 25, 50\}$
Initial network structure	$\iota \in \{\text {empty}, \text {full}\}$	$\iota \in \{\text {empty}, \text {full}\}$
Proportion of ties to evaluate per time step	$0 < \phi \le 1$	$\phi = 0.4$

#### Utilities

In accordance with the literature on the CM, we consider relatively low net benefits of direct connections and relatively high net benefits of connections at distance 2 ($\alpha - c < \beta$). That is because net benefits of direct connections that are higher than benefits of connections at distance 2($\alpha - c > \beta$) inevitably result in fully connected networks. To distinguish settings in which indirect ties are valued relatively highly (e.g., professional networks providing access to resources), and settings in which indirect ties are valued relatively lowly (e.g., friendships likely to result in triadic closure), we compare two parameter settings: $\beta = 2$ and $\beta = 8$. To limit the change in benefits to a single varied parameter, we neglect changing benefits due to infections ($\kappa = 1.0$, $\lambda = 1.0$).

To investigate overall lowered social value of direct relations ($B_{i} - C_{i}$), we vary costs for infected connections. One setting without increased costs ($\mu = 1.0$) and one with increased costs for infected ties ($\mu = 1.5$). This translates to situations in which infected individuals typically recover alone while staying independent and situations that require support from others, because infected individuals cannot master their everyday life alone (e.g., requiring somebody to get groceries). Costs to maintain connections is set to a constant value ($c = 9$) to accomplish two things. First, we do not obtain fully connected networks, because net benefits for direct connections ($10 - 9 = 1$) are smaller than benefits of connections at distance 2 for both settings. Second, net benefits of direct and indirect connections combined vary greatly depending on benefit of connections at distance 2 ($\beta$).

Diseases in the current implementation of the CIDM are considered generic. Thus, they may range from mild ($\sigma = 2$) through moderate ($\sigma = 10$) to severe ($\sigma = 50$). The probability to get infected per contact and time step is constant at $\gamma = 0.1$. We assume that risk perceptions with regard to disease severity and susceptibility coincide ($r_{\sigma } = r_{\pi } = r$). Further, we assume agents of the same population to perceive risks equally. This form of homogeneity applies more to social context and equal distribution of information rather than individual differences. Consequently, we compare three different types of populations with regard to risk perception. First, agents perceiving risk to be lower than actual risk (*low risk*; $r_{\pi } = r_{\sigma } = r = 0.5$). Second, agents perceiving risk realistically (*realistic risk*; $r_{\pi } = r_{\sigma } = r = 1.0$). Third, agents perceiving risk to be higher than actual risk (*high risk*; ($r_{\pi } = r_{\sigma } = r = 1.5$). Infected agents in the CIDM require a fixed number of time steps to recover ($\tau = 10$). Simulation test runs showed that this combination of settings allows to prevent all agents to get infected or to disconnect from infectious others immediately after introduction of the disease into the network, and consequently to create strong variations of network and disease dynamics.

#### Network

We simulated populations of 10, 15, 20, 25, and 50 agents. Although larger populations are of empirical interest for disease dynamics, the CM typically describes social contexts of limited group size. Furthermore, pilot simulations have shown that results for larger network sizes do not differ qualitatively from networks with 50 agents, due to increasingly large degrees of agents in larger networks. Additionally, we differ between two starting conditions with regard to network density: empty ($\iota =$ empty, with $g_{ij} = 0$ for all *ij*) and fully connected networks ($\iota =$ full, with $g_{ij} = 1$ for all $i \ne j$ and $g_{ii} = 0$). This ensures that pairwise stability emerges from networks with different densities. Finally, we set the number of existing or potentially new ties an agent may evaluate per time step to $40\%$ of the population ($\phi = 0.4$). This is done to account for the idea that people do not always have full control over their social relations (a train conductor at work, parents picking up their children from daycare) and the possibility of disease transmissions from asymptomatic and thus unrecognized infectious persons. Again, this holds true for every agent, thus referring to social context (e.g., work environment) and leveling out individual differences within the population.

We systematically combined all previously defined parameter values, resulting in a total of 360 different parameter combinations. Further, we ran 100 simulations for each parameter combination, resulting in a total number of 36,000 simulation runs.

### Software

The simulation was programmed using the Java 8 programming language and the GraphStream 1.3 library [65] for graph handling. The complete code, including an executable program and an easy-to-use graphical user interface, is freely accessible under the GPLv3 license [66].

## Data and analysis

We log detailed network and disease information for each agent and time step of each simulation run. To understand how network dynamics shape the course of epidemics, we use regression analyses of simulation data [cf., 67, 68] with regard to: (i) the proportion of recovered agents at the end of each simulation (*final size of the epidemic*, or short: *final size*) and (ii) the number of time steps until all once infected agents have recovered (*duration of the epidemic* or short: *duration*). To disentangle how much of our results are driven by parameter settings and how much is due to stochasticity of the simulation, we use two-level random-intercept regressions (level 2: 360 parameter combinations; level 1: 100 simulation runs). Random elements in the simulation are: (i) agent who is initially infected; (ii) order of agents that change their disease states on network connections; (iii) which network connections are considered and in which order. Furthermore, we create four models, each based on the previous model: (i) *Empty*, (ii) *CIDM effects*, (iii) *Network effects*, and (iv) *Interaction effects*. The Empty model shows how much unexplained variance is due to stochasticity between and within the same parameter settings. It further allows to compare the reduction of unexplained variance when controlled for the varied CIDM model parameters (CIDM effects model). The Network effects model further controls for network measures (network density, degree of patient-0), while the Interaction effects model adds statistically significant interaction effects of all previously described main effects. Data points for regression analyses are single simulation runs.

According to Long [69], logit (and probit) models are most suitable when the dependent variable is a proportion of a binary response. Final size corresponds to this requirement as it describes the proportion of agents that have been infected with the disease and those that have not. The statistical model estimates how the logit of final size can be approximated linearly by a combination of the parameters. Further, we use two-level random-intercept linear regression to analyze the model with regard to duration of the epidemic.

We consider linear relationships for all varied parameters, because they are either simulated with only two different values ($\beta$, $\mu$, $\iota$), or separate model parameters for more than two values ($\sigma$, *r*) and manipulation of parameters ($N^{x}$) did neither improve model fit ($\ell$) nor substantively reduced unexplained variance ($\rho$). Interactions are constructed using grand mean centering, while maximum likelihood is used to estimate parameters. To allow easier interpretation of model coefficients, we rescale disease severity ($\frac{\sigma }{50}$) and network size ($\frac{N}{50}$).

For visual inspection of the progression of epidemics we generate SIR model plots, a method commonly used to display the temporal development of compartments during epidemics. We plot only time steps relevant for the epidemics. That is, rather than showing all simulated time steps, plots begin 10 time steps prior to the introduction of a disease (epidemic stage) and display 80 time steps in total. Next to showing the dynamics over all simulations, we also report the dynamics for networks in which agents change relations as a consequence of the introduction of a disease next to the dynamics for simulation runs in which this does not happen. Although we never actively turned off the option to change ties, there are quite some parameter constellations in which actors do not have a reason to change ties. A comparison between networks with and without tie changes therefore allows a clearer picture of the effects of network dynamics on epidemics.

To relate the network and disease dynamics to some overall network characteristics, we compute several characteristics of networks at different moments during the simulation. Density is computed as the proportion of all possible connections present in the network [70 p. 101 ff.]. Clustering is calculated as the average proportion of closed triads over the number of all possible triads per agent [71]. Finally, closeness is computed by reversing the normalized average distance between any two nodes in the network; network size (*N*) is imputed as distance for pairs of nodes that are not connected through any path in the network [72, p. 163]. For analyses we use R version 3.6.0 [73] with additional packages *lme4* [74] for logit regressions, *texreg* [75] for export of results, and *ggplot2* [76] for data visualization.

## Results and discussion

### Model behavior

**Fig. 1:**
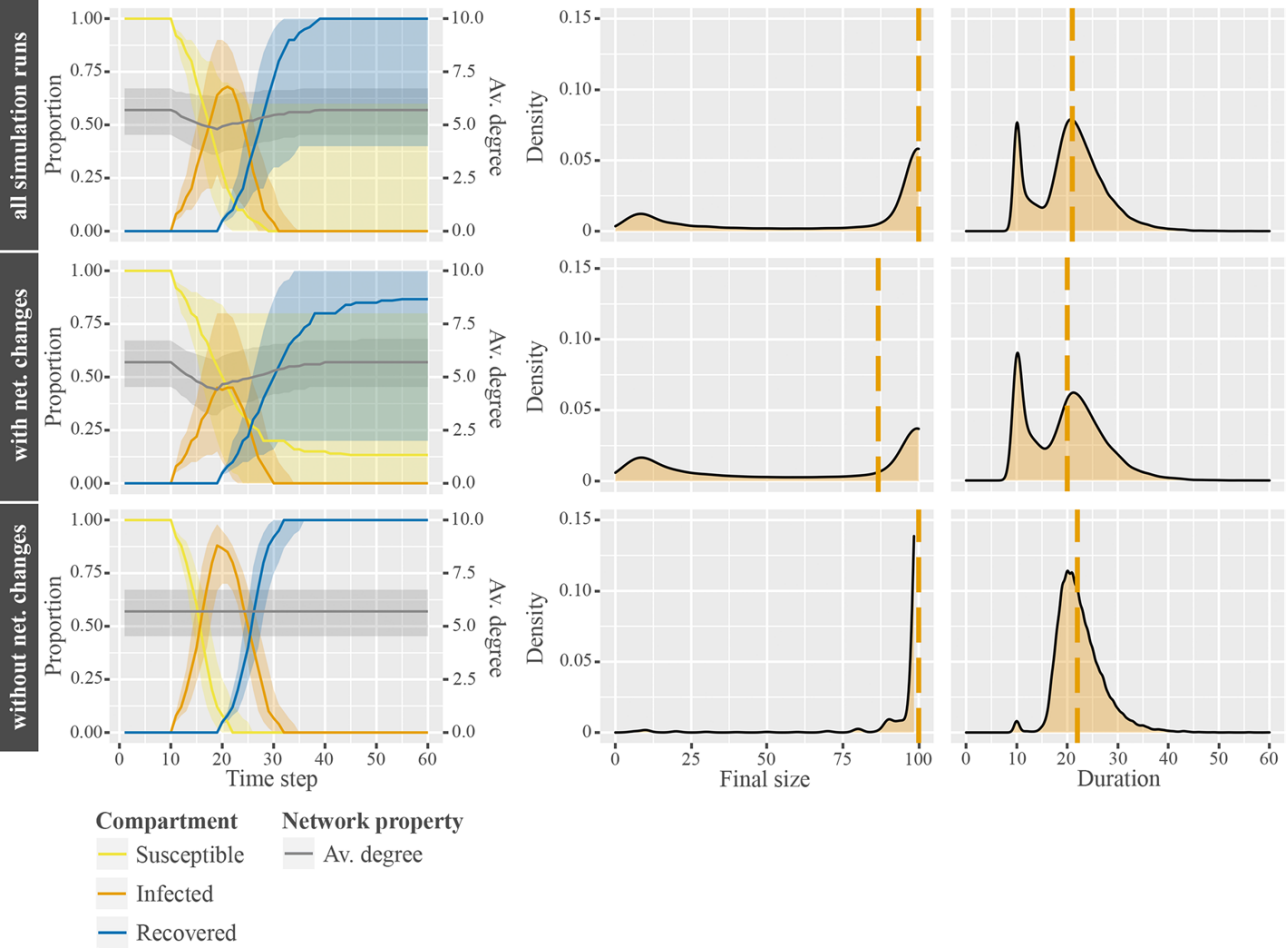
Disease dynamics in networks with and without network changes. Column 1 shows plots for the median proportional changes of agents according to disease states (yellow, orange, and blue lines), changes in median average network degree (gray lines), and interquartile ranges (shaded areas around median lines) over time. Column 2 shows the distribution of final size of epidemics. Column 3 shows the distribution of duration of epidemics. Row 1 contains the data of all simulation runs combined. Row 2 shows the data for simulation runs with network changes. Row 3 shows the data for simulations without network changes. The dashed orange line indicates the median of the respective distributions

Figure 1 shows a clear interaction between social network and infectious disease dynamics, especially when comparing simulation runs with network changes (row 2) to simulation runs without network changes (row 3). Column 1, for example, shows the progression of epidemics over time. While in both cases (rows 2 and 3), after the introduction of the disease at time step 10, the proportion of infected agents increases and peaks at approximately time step 21 (orange line), the peak for simulation runs with network changes is significantly lower. At the same time, average degree drops from about 6 to below 5 ties per agent in row 2, while average degree remains stable in row 3. Considering that networks were pairwise stable before the initial infection, the average number of 3.41 network changes per agent (sum of dissolution and creation of ties) is caused and mainly driven by the presence of infectious agents. Additionally, we observe that even more network changes were initiated by agents trying to connect to others (on average 18.00 tie requests per agent). These tie requests, however, were accepted only in $9\%$ of the cases. This is a vast difference to the network initialization stage prior to the epidemics, where $40\%$ tie requests were accepted from an average of 7.97 tie requests per agent.^4^ Over time, infected agents recover, resulting in decreases in the infected compartment, increases in the recovered compartment, and a restoration of degree to the pre-epidemic state in row 2.

Furthermore, column 2 of Fig. 1 reveals a bimodal distribution of final size in simulation runs with network changes (row 2). On the left a comparably small peak shows the simulation runs with only a single agent infected. On the right a large peak shows the simulation runs with all agents infected. It follows, that in most simulations the epidemic either died out immediately or spread across the entire network. This bimodal effect is the driving force behind the large width of interquartile ranges in row 2, especially for the final proportions of susceptible and recovered agents. In simulation runs without network changes (row 3), however, we observe virtually no variance in final size, as the vast majority has all agents infected at some point.

Considering the average duration of epidemics, we can observe a corresponding bimodal effect for simulation runs with network changes (column 3, row 2). The left peak shows the recovery time for a single infection. The right peak shows the average duration of epidemics for final sizes of close to $100\%$. Note that the left peak is more distinct for duration than final size, because one single infection requires exactly 10 time steps for recovery, while final size is a proportion depending on network size.

These data suggest that, on an individual level, agents succeed under certain conditions to avoid potential harm of diseases by distancing themselves from infected contacts during an epidemic. That is, agents cut ties in order to move into low-risk network positions, unless social benefits outweigh the potential harm of an infection. On the population level, cutting ties ultimately leads to smaller and shorter epidemics. In the following, we investigate which CIDM and network parameters drive the variances in these two epidemic measures.

### CIDM parameters

Tables 2 and 3 show the effects of CIDM parameters on final size and duration of epidemics, respectively. The *Empty* regression model shows that in our following elaborations we will be able to attribute $69\%$ of the overall variance in final size and $31\%$ of variance in duration (see intraclass correlation coefficients $\rho$) to variation of parameters, while the remainders are caused by the stochastic elements of the simulation.

**Table 2:** Two-level random-intercept logistic regression of final size of epidemics

	Empty	CIDM effects	Network effects	Interaction effects
Fixed effects (observed effects)				
Intercept	**2.27** (0.15)	**2.29** (0.08)	**2.29** (0.08)	**2.59** (0.07)
Main effects				
I. CIDM parameters				
Benefit distance 2 ($\beta$)		**0.25** (0.02)	** 0.25** (0.02)	**0.23** (0.02)
Cost increase for infected ties ($\mu$)		− **4.97** (0.30)	− **5.00** (0.30)	− **5.30** (0.21)
Disease severity ($\frac{\sigma }{50}$)		− **1.87** (0.17)	− **1.87** (0.17)	− **1.75** (0.11)
Risk perception (*r*)		− **2.74** (0.18)	− **2.75** (0.18)	− **3.16** (0.13)
Network size ($\frac{N}{50}$)		** 5.56** (0.29)	**4.66** (0.37)	**6.23** (0.38)
II. Network properties (start of epidemics)				
Density (${\text{den}}_{\text{start}}$)			− **7.68** (1.00)	− **7.33** (0.97)
Degree of patient-0 (${\text{deg}}^{0}_{\text{start}}$)			**3.86** (0.32)	**6.46** (0.51)
Interaction effects				
Benefit distance 2 ($\beta$) $\times$ cost increase for infected ties ($\mu$)				**0.52** (0.06)
Cost increase for infected ties ($\mu$) $\times$ disease severity ($\frac{\sigma }{50}$)				**3.16** (0.44)
Cost increase for infected ties ($\mu$) $\times$ risk perception (*r*)				**6.40** (0.49)
Disease severity ($\frac{\sigma }{50}$) $\times$ risk perception (*r*)				− **2.04** (0.27)
Risk perception (*r*) $\times$ network density (${\text{den}}_{\text{start}}$)				**9.44** (1.49)
Network size ($\frac{N}{50}$) $\times$ degree of patient-0 (${\text{deg}}^{0}_{\text{start}}$)				**13.80** (2.10)
Random effects (unobserved effects; Intercept)				
$s^{2}$	7.33	1.44	1.41	0.47
Log likelihood ($\ell$)	− 12,079.15	− 11,814.47	− 11,734.67	− 11,581.32
Intraclass correlation ($\rho$)	0.69	0.31	0.30	0.13
Observations	36000	36000	36000	36000
Groups: parameter combinations	360	360	360	360

Bold coefficients are significant at $p<0.001$, others are not significant ($p>0.05$), SEs in parentheses

**Table 3:** Two-level random-intercept linear regression of duration of epidemics

	Empty	CIDM effects	Network effects	Interaction effects
Fixed effects (observed effects)				
Intercept	**20.66** (0.20)	**20.66** (0.17)	**20.66** (0.16)	**21.23** (0.19)
Main effects				
I. CIDM parameters				
Benefit distance 2 ($\beta$)		**0.36** (0.06)	**0.36** (0.05)	**0.36** (0.04)
Cost increase for infected ties ($\mu$)		− **3.40** (0.66)	− **3.42** (0.63)	− **3.42** (0.44)
Disease severity ($\frac{\sigma }{50}$)		− **1.82** (0.39)	− **1.81** (0.38)	− **1.81** (0.26)
Risk perception (*r*)		− **2.80** (0.41)	− **2.81** (0.39)	− **2.81** (0.27)
Network size ($\frac{N}{50}$)		**2.05** (0.59)	− **3.20** (0.70)	− 0.11 (1.15)
II. Network properties (start of epidemics)				
Density (${\text{den}}_{\text{start}}$)			− **26.87** (1.96)	− **19.93** (3.20)
Degree of patient-0 (${\text{deg}}^{0}_{\text{start}}$)			**3.30** (0.62)	**3.31** (0.62)
Interaction effects				
Benefit distance 2 ($\beta$) $\times$ cost increase for infected ties ($\mu$)				**1.05** (0.15)
Benefit distance 2 ($\beta$) $\times$ network size ($\frac{N}{50}$)				− **1.06** (0.13)
Cost increase for infected ties ($\mu$) $\times$ density (${\text{den}}_{\text{start}}$)				− **38.17** (5.00)
Disease severity ($\frac{\sigma }{50}$) $\times$ risk perception (*r*)				− **4.16** (0.64)
Disease severity ($\frac{\sigma }{50}$) $\times$ network size ($\frac{N}{50}$)				**6.48** (0.93)
Risk perception (*r*) $\times$ network size ($\frac{N}{50}$)				**8.20** (0.96)
Network size ($\frac{N}{50}$) $\times$ network density (${\text{den}}_{\text{start}}$)				**32.97** (9.35)
Random effects (unobserved effects)				
$s^{2}$	13.66	9.58	8.63	3.96
Log likelihood ($\ell$)	− 113,544.27	− 113,481.97	− 113,388.50	− 113,274.74
Intraclass correlation ($\rho$)	0.31	0.24	0.22	0.11
Observations	36000	36000	36000	36000
Groups: parameter combinations	360	360	360	360

Bold coefficients are significant at $p<0.001$, others are not significant ($p>0.05$), SEs in parentheses

The *CIDM effects* regression model reveals that higher benefits of indirect connections ($\beta$) result in increases of final size and duration of epidemics. That is because higher social benefits may outweigh the potential harm of infections, and thus agents are more likely to stay connected. This causes more agents to get infected. Furthermore, the results of our model suggest that higher numbers of infected require more time for all agents to recover. Note that the longer duration is not an inevitable consequence of larger final sizes, as more intense epidemics may create many simultaneously infected agents without significant impact on duration.

Increasingly severe diseases, both objectively ($\sigma$) and subjectively perceived (*r*), and higher costs for infected direct ties ($\mu$) create smaller final sizes and shorter duration of epidemics. That is because more severe diseases and higher costs for infected ties reduce the net benefit of a relationship to such a degree that potential costs of infections or existing costs exceed the social gains of the relation to an infected agent. Consequently, more agents disconnect from their partner, causing less agents to be infected, which in turn requires less time steps for the disease to disappear.

Finally, larger networks result in higher proportions of infected agents and longer lasting epidemics. The effect on duration is straightforward, as more consecutively infected agents require more time to recover. The effect on final size, however, is not intuitively comprehensible on its own. Thus, we consider the interplay with additional network properties in the following section.

### Network properties

By adding network density and degree of the first infected agent at the time when the disease is introduced into the network, the *Network effects* regression model reveals larger final sizes of epidemics when: (i) network size increases, (ii) the network is less dense, or (iii) patient-0 has more ties. The positive effect of higher degrees for patient-0 can be explained with statistical probabilities. That is, the more connections an infected agent has, the more likely the disease is being transmitted to other agents. The other two effects require a closer look.

Table 4 shows a negative relationship between network size and density. Hence, larger networks have a lower proportion of actual connections compared to potential connections. Network size, however, has a positive effect on average degree. In other words, when networks grow larger the overall number of connections increases while fewer potential connections are formed. Applying the previous argument of statistical probabilities in context of average degree, we observe that a larger total number of connections increases the chance for a disease to spread. Thus, the effect of network size is related to the effect of average degree, which has a decisive impact on the final size of the epidemic.^5^

**Table 4:** Mean density, average degree, final size, and duration of epidemics per network size

Network size	Density	Av. degree	Final size	Duration
10	0.404 (0.027)	3.632 (0.244)	56.987 (38.023)	18.406 (7.788)
15	0.338 (0.016)	4.736 (0.230)	66.636 (38.890)	20.434 (7.552)
20	0.299 (0.012)	5.688 (0.230)	73.880 (37.272)	21.481 (6.803)
25	0.273 (0.009)	6.545 (0.227)	80.395 (33.792)	21.999 (5.999)
50	0.204 (0.004)	9.994 (0.206)	95.855 (18.140)	20.971 (3.922)

**Fig. 2:**
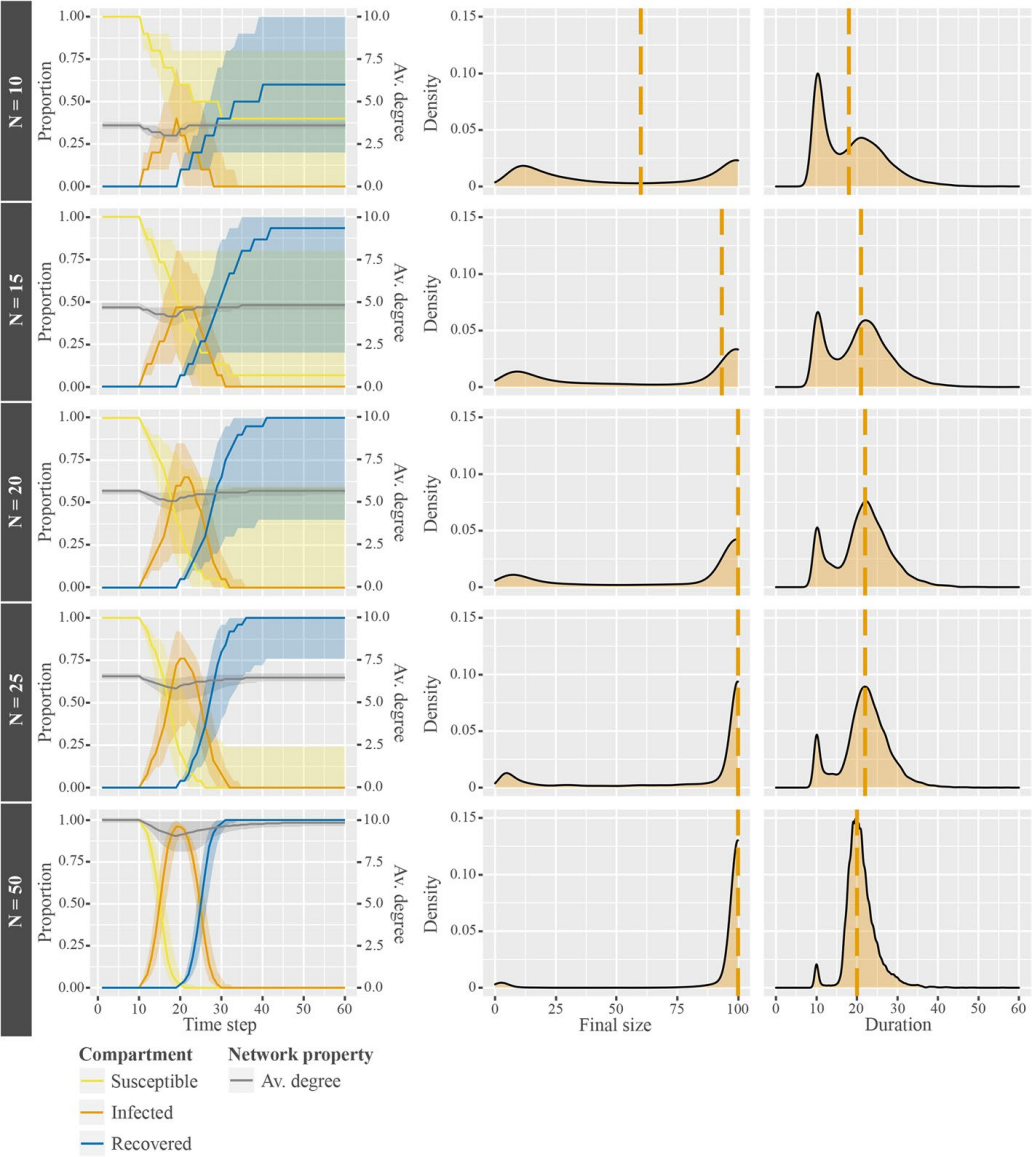
Disease and network dynamics by network size. Column 1 shows plots for the median proportional changes of agents according to disease states (yellow, orange, and blue lines), changes in median average network degree (gray lines), and interquartile ranges (shaded areas around median lines) over time. Column 2 shows the distribution of final size of epidemics. Column 3 shows the distribution of duration of epidemics. Rows show data split by network size. The dashed orange line indicates the median of the respective distributions

Figure 2 reveals that epidemics are larger for larger network sizes, despite similar behavioral reactions of the agents. Column 1, for example, shows that the reduction of average degree during the epidemics is similar for all network sizes (about 1 degree on average), while the overall average is at considerably different levels (3.632 for $N = 10$, 9.994 for $N = 50$). As a result, more connections remain in larger networks providing more potential transmission routes. Consequently, in large networks agents simply cannot dissolve enough ties to distance themselves from the disease sufficiently resulting in almost all agents getting infected in every simulation run. A comparison of disease dynamics between simulation runs with and without network changes during epidemics (Figs. 5 and 6 in Appendix) shows that the differences of final size and duration of epidemics between different network sizes is largely driven by agents cutting ties and not merely an effect of higher average degrees in larger networks.

Furthermore, we observe a negative effect of network size on the duration of epidemics when combined with density and degree of patient-0 (*Network effects*, Table 3). That is, epidemics last longer when: (i) network size decreases, (ii) the network is less dense, or (iii) patient-0 has more ties. At first sight, these results may not seem plausible. In fact, networks with 10 agents have the lowest average duration of epidemics (18.406 time steps, see Table 4), while networks with 25 agents have the longest lasting epidemics on average (21.999 time steps). The largest simulated networks with 50 agents, however, show a decrease in average duration of epidemics (20.971 time steps). This non-linear effect for the duration of epidemics is explained by two opposing factors: first, the larger a network gets (assuming constant average degree) the more time is needed to infect the entire network. Second, the larger the network the larger the average degree (see Table 4). As discussed earlier, larger average degrees result in higher chances to spread a disease. Consequently, larger average degrees increase the likelihood for disease transmissions per time step and therefore facilitate faster disease spread than lower average degrees. Networks up to 25 agents approximate the tipping point of these opposing effects: smaller networks are large enough to slow down disease spread stronger than average degree speeds it up.

Note that the relation between network size, network density, and average degree is an artifact of the CM. Although implications of degree scaling with network size become more unrealistic the larger the networks get, it illustrates that dynamics can significantly change with network size. Furthermore, it shows the importance of choosing appropriate network formation models for specific model cases.

### Interaction effects

The *Interaction effects* regression models help to understand the interdependence between disease and network dynamics. They were selected exploratively based on their effect on improvement of model fit ($\ell$) and explained variance ($\rho$). As these effects are not all intuitive, we take a closer look at two representative interactions that affect both final size and duration of the epidemics: (i) benefit of connections at distance 2 ($\beta$) $\times$ cost increase for infected ties ($\mu$), and (ii) disease severity ($\frac{\sigma }{50}$) $\times$ risk perception (*r*).

**Fig. 3:**
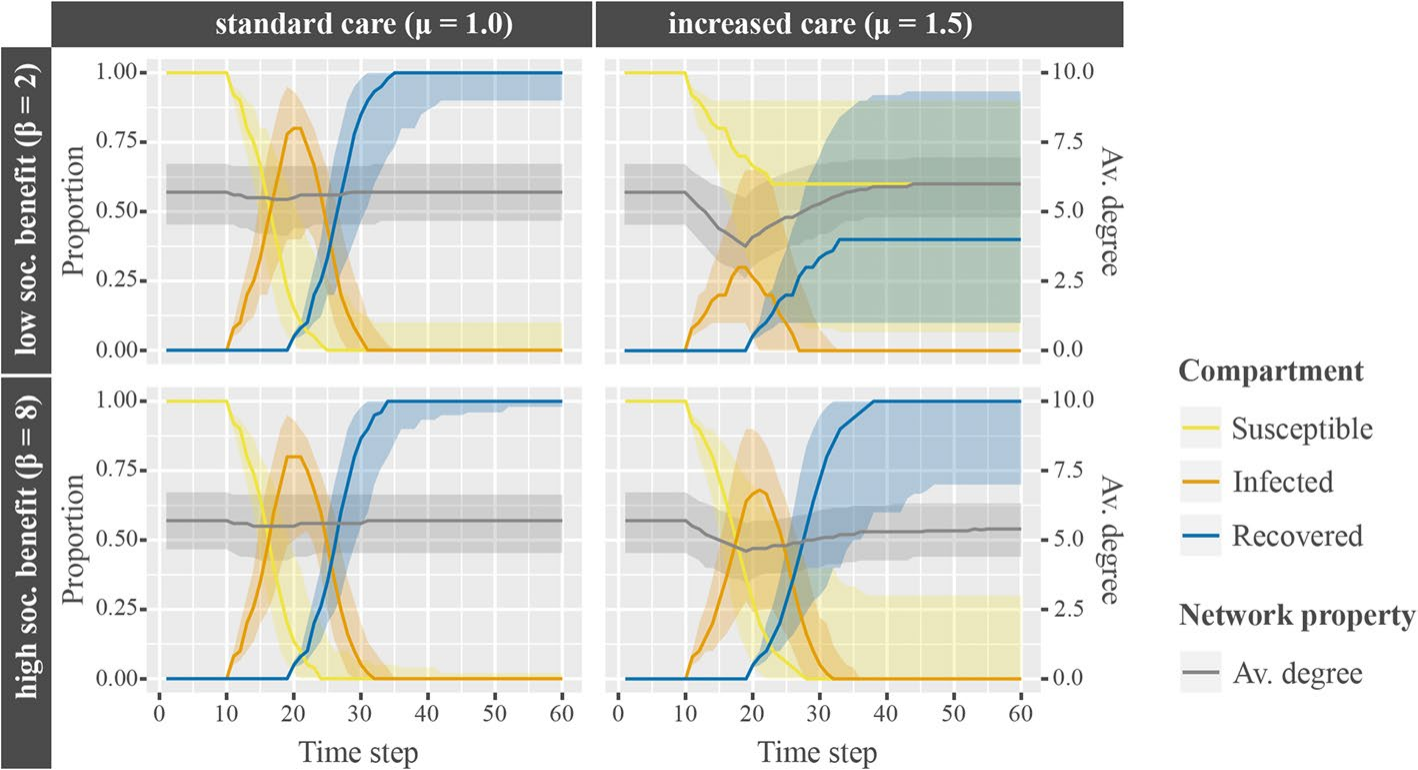
Interaction of costs for infected ties and value of indirect connections. Interaction of increased costs for infected ties ($\mu$, columns) and value of indirect connections ($\beta$, rows) on the median proportional changes of agents according to disease states (yellow, orange, and blue lines), changes in median average network degree (gray lines), and interquartile ranges (shaded areas around median lines) over time

As discussed before, increased benefits at distance 2 ($\beta$) show positive effects, while cost increases for infected ties ($\mu$) show negative effects on final size and duration of epidemics. This is shown again in Fig. 3. The bottom left plot shows that for parameter combinations with standard care costs and high social benefits for connections at distance 2, epidemics are typically large (${\text{Median}}= 100\%$) with long duration (${\text{Median}}= 22$ time steps). Parameter combinations that differ from the bottom left in one parameter only (increased care costs for infected ties, bottom right; low social benefit, top left), show similar final sizes and average duration. Parameter combinations with both increased care costs for infected ties and low social benefits (top right), however, show significantly smaller final sizes (${\text{Median}}= 40\%$) and shorter duration of epidemics (${\text{Median}}= 17$ time steps).

The average degrees of the networks (gray lines) show that highly valued connections at distance 2 (bottom row) create low dissolution of ties independent of whether the sickness requires more care for an infected connection. In contrast, lower valued connections remain only intact when care for the sick requires no extra costs (top left). Consequently, parameter combinations with low social benefits and increased costs for infected ties (top right) cause many agents to disconnect and thus successfully distance themselves from the disease once it enters the network.

**Fig. 4:**
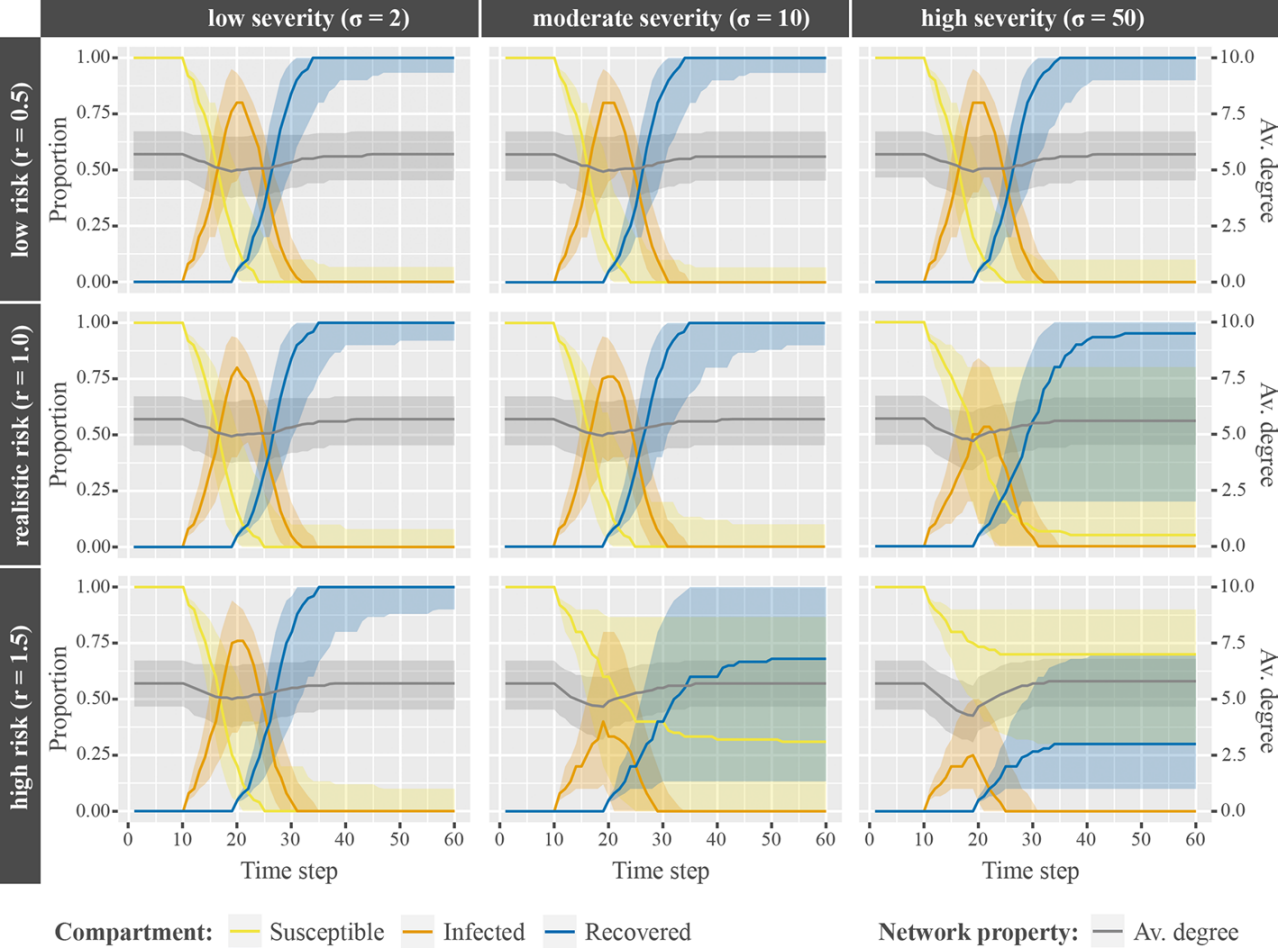
Interaction of disease severity and risk perception. Interaction of disease severity ($\sigma$, columns) and risk perception (*r*, rows) on the median proportional changes of agents according to disease states (yellow, orange, and blue lines), changes in median average network degree (gray lines), and interquartile ranges (shaded areas around median lines) over time

**Fig. 5:**
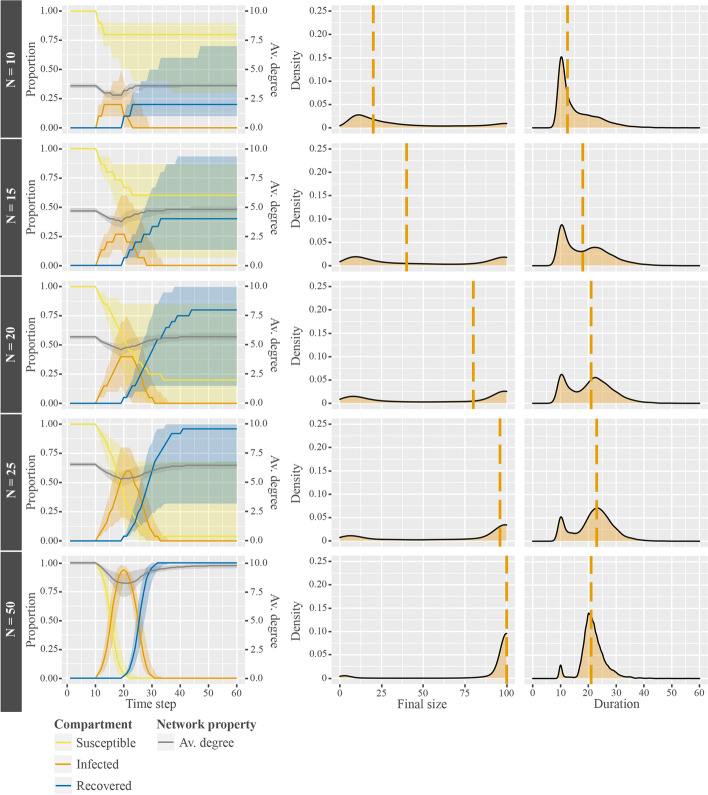
Disease dynamics by network size in network with network changes. Simulation runs with network changes: interplay of network and epidemic dynamics by comparison of median proportions of disease states and average degree over time (column 1), distribution of final size of the epidemic (column 2), and distribution of duration (column 3), divided by network size. The dashed orange line indicates the median of the respective distributions

**Fig. 6:**
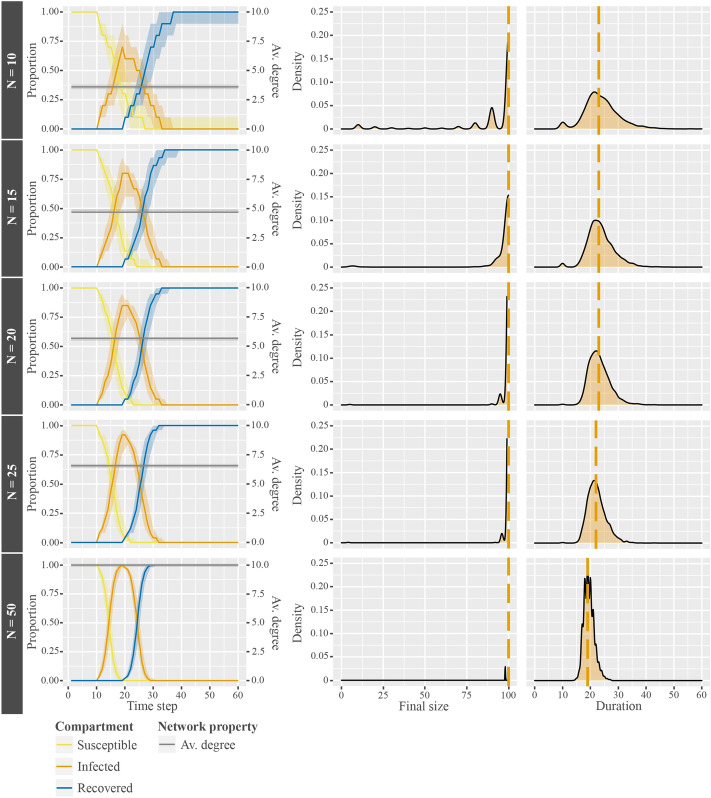
Disease dynamics by network size in network with network changes. Simulation runs without network changes: interplay of network and epidemic dynamics by comparison of median proportions of disease states and average degree over time (column 1), distribution of final size of the epidemic (column 2), and distribution of duration (column 3), divided by network size. The dashed orange line indicates the median of the respective distributions

A similar picture is drawn for disease severity ($\sigma$) and risk perception (*r*), where both parameters show negative main effects on final size and duration of epidemics individually. Again, these effects are stronger in combination than individually (Fig. 4). Parameter combinations with high-risk populations and varied disease severity (top row) show equal average final sizes (${\text{Median}}= 100\%$) and similar duration of epidemics (${\text{Median}}=[21, 22]$ time steps). The same applies for mild diseases and various risk perceptions (left column; final size: ${\text{Median}}= 100\%$; duration: ${\text{Median}}=[21, 22]$ time steps). Parameter combinations with low-risk populations and highly severe diseases (bottom right), however, have the lowest final size (${\text{Median}}= 30\%$) and duration of epidemics (${\text{Median}}= 15$ time steps). Again, the decisive factor for these results is average network degree: agents disconnect more if they perceive risk to be high *and* diseases are severe (bottom right) compared to parameter combinations with agents perceiving risk to be low (top row) *or* mild diseases (left column) alone.

Both depicted interactions demonstrate another highly important effect: an underlying two-way interaction between the social network and the disease. First, the higher the average degree the more serious the epidemic. Second, the more serious the (subjective) consequences of a disease (here: increased costs for infected ties or perceived severity) the stronger the behavioral reaction and thus the lower the average degree.

Further, Fig. 4 allows looking at the effect of more subtle changes of tie dissolution. Consider the effect of average degree on final size of the epidemic in parameter combinations with highly severe diseases and differing risk perceptions. First, populations with agents perceiving the risks realistically (center right), and second, populations with agents perceiving high risks of infections (bottom center). We see that small changes of average degree (from ${\text{Median}}= 4.53$ to ${\text{Median}}= 4.13$) create large differences in final size (from ${\text{Median}}= 95\%$ to ${\text{Median}}= 30\%$), peak height (from ${\text{Median}}= 55\%$ to ${\text{Median}}= 25\%$), and duration of epidemics (from ${\text{Median}}= 31$ to ${\text{Median}}= 25$). This enables two important insights: first, epidemics are highly sensitive to social distancing in dynamic social networks. Second, even small changes on the individual level may have major consequences on the large scale.

## Conclusion and implications

The role of social networks in the spread of infectious diseases has gained more attention in recent years. Networks are commonly considered static or network dynamics are modeled as random processes devoid of health behavioral foundations [see, 7, 15]. We know, however, that people deliberately seek to distance themselves from diseases in social networks [3, 8, 61], or are instructed to distance themselves from others at times of increased risk of infection [77–79].

To address this, we propose the *Networking during Infectious Diseases Model* (*NIDM*), a highly adaptable steppingstone model for the co-evolution of social networks and infectious diseases. This model combines theories of social network formation from sociology, infectious diseases from epidemiology, and individual health behavior from social psychology. We argue that networking behavior in the context of infectious diseases is a trade-off between the social well-being a contact creates, the costs required to maintain a social tie, and the potential physical harm infectious contacts create. Furthermore, we created a specific model case of the NIDM based on the truncated version of the *Connections Model* [*CM*; 41, 64], a model typically describing social contexts of limited group size, including a multi-agent simulation in which agents deliberately distance themselves from infectious contacts in social networks whenever the potential harm of getting infected through a tie outweighs its social benefit.

Based on our simulations, we gain a number of theoretical insights: we find average degree as a major determinant for epidemic size and duration of epidemics. That is, larger numbers of connections provide more opportunities for disease spread, thus causing more agents to get infected and shorter epidemics. Furthermore, we see a highly interdependent process between the properties of agents, diseases, networks, and epidemics: the higher the (perceived) risks of a disease, the lower the net benefit of a tie, the stronger the social distancing, and consequently the lower the epidemic size. While these results align with our expectations, we gained more surprising insights thanks to our novel network approach. First, networks with benefits for social connections that induce many ties per agent, provide large numbers of potential transmission routes, and consequently allow the disease to travel faster. Second, higher costs of maintaining ties with infected others reduce final size of epidemics only when benefits of indirect ties are relatively low. Third, we find that small changes in social behavior have large effects on the epidemic, which may be decisive for whether the outbreak of a disease turns into an epidemic or not.

Our theoretical outcomes are in line with empirical observations and other model studies, inter alia, from Ahmed et al. [61] and Danon et al. [80]. The former is a review of empirical studies that showed reduction in the number of infections and lower epidemic peaks due to workplace social distancing. The latter is an epidemic network model considering the social structure of populations suggesting that high average degrees result in increases of final size of epidemics. Similar to Poletti et al. [20], our results suggest that even small reductions in contact numbers can reduce epidemic impact. While in line with the results of Leung et al. [15] (strong stochastic rewiring creates small epidemic sizes, moderate stochastic rewiring creates large epidemic sizes), our model can provide a more explicit theoretical mechanism for their findings. More precisely, our model provides a tool to improve models of infectious diseases by adding network formation processes based on theories from sociology and health psychology, rather than using purely stochastic rewiring processes or artificially lowering transmissibility [3, 7, 15]. Additionally, our model has proven to be useful to reveal previously unknown and less intuitive mechanisms (e.g., higher costs of maintaining ties with infected others reduce final size of epidemics only when benefits of indirect ties are relatively low). Furthermore, we contribute to solving the issue that neglect of health behaviors may create mismatches between observed transmissibility of diseases and epidemic sizes of model predictions [7]. Although some of our theoretical insight could help to understand earlier empirical findings, the empirical validity of our new predictions needs tailored empirical testing in subsequent studies.

It is most important to stress that our general model, the NIDM is a highly adaptive steppingstone to capture the basic characteristics of the co-evolution of dynamic social networks and infectious disease dynamics, which allows many opportunities for extensions and specifications in each part of the model. First, social utilities, and thus resulting network structures, can be modeled with regard to context (social, disease, cultural). HIV, for example, is typically modeled using sexual contact networks (benefits for direct contacts only) that allow great control over single contacts (large $\phi$ parameter). Further, social maintenance costs ($C_{i}$) can be extended with moral costs for infected individuals to infect others. Second, diseases can be defined according to their objective risks (lethality, stigma, financial), disease states, and treatments. As there is no cure yet for HIV, infected individuals do not recover but remain infected until death. Adoption of preventive behaviors (e.g., intake of PrEP) lowers or may even eliminate the probability of transmission. Third, subjective perception of health risks ($r_{\pi }$, $r_{\sigma }$) can differ according to the availability of information or risk perceptions, an effect utilized by awareness-raising campaigns.

It is furthermore important to stress that any specific model case of the NIDM will come with limitations depending on the modeling choices made. While our current model case, for example, the *Connections during Infectious Diseases Model* (*CIDM*), provides an interesting interplay of social and infectious disease dynamics, the CM is designed and typically used to describe small networks. Our simulations suggest that the CM is not feasible for network sizes of 50 and above, as average degree grows with network size ultimately causing all agents to get infected. One approach to realize larger plausible social networks could be the extension of the CM with an explicit saturation term for the number of social relations. Another approach is to design a different baseline utility to describe the utility of social relations for a specific context. Especially the choice of social contexts need to be considered carefully. In friendship networks, for example, we would expect to observe triadic closure, and thus increasing network density and clustering, when indirect contacts are beneficial. Thus, professional networks, as studied by Ahmed et al. [61], seem more plausible as an application for the CM as baseline utility, as indirect contacts may provide benefit through access to rare information unavailable through direct contacts, without the same likelihood for closing the triad. Further, while it seems plausible that agents assess health risk on local information, the assumption that agents have complete knowledge about the contacts of potential new network partners is not quite realistic. Still, we think that in a realistic process of network formation, people investigate into relations before establishing certain ties. Our assumption can also be interpreted as a simplification of this process, assuming that people indeed are able to obtain this information if they want to establish a relation. Alternatively, one could investigate alternative assumptions, such as random creation of new ties followed by dissolution if the relation is not satisfactory. Furthermore, due to low value and variance of network clustering in the CIDM, we cannot make inferences on how networks with dynamic clusters affect the course of epidemics. Empirically, we know that social networks are typically highly clustered (a property that hardly emerged in our networks) while having small average path lengths (*small worlds*) and that these properties can facilitate disease spread [56, 71]. Furthermore, people differ in how they perceive risks and translate this into action. It follows, that an outbreak in a cluster of individuals perceiving risks to be high may prevent an epidemic, while a few low-risk individuals in a high-risk population may accelerate disease spread.

In conclusion, our theoretical insights suggest that modeling health behavior explicitly in network models of disease spread can help to gain a broader theoretical foundation and deeper insights into the co-evolutionary processes of dynamic social networks and infectious diseases. Furthermore, the steppingstone model we propose—the *Networking during Infectious Diseases Model (NIDM)*—has proven to be a valuable tool for such an undertaking.

## Data Availability

The datasets, the Java 8 source code to generate the data (including an executable program and an easy-to-use graphical user interface), and the R scripts to analyze the data during the current study are available under the GPLv3 license in the GitHub repository, https://github.com/hnunner/NIDM-simulation (version: v4.1.0., commit: f17e0b0, DOI: 10.5281/zenodo.4290115).

## Appendix

Figures 5 and 6 show the results of Fig. 2 divided between simulation runs with network changes and without network changes. A comparison of the two figures shows that epidemics in networks where ties are being cut (Fig. 5) have a significantly lower final size and mostly shorter duration across all network sizes. Furthermore, epidemics without network changes show hardly any variance in final size or duration (Fig. 6). Variance in epidemic measures is therefore not merely an effect of average degree in different network sizes, but sensitive to network changes of agents trying to distance themselves from the disease. An integrated discussion of these results can be found in section Network properties.

Table 5 shows descriptive statistics over the whole data for epidemics, network dynamics during and before epidemics, and network measures at the last time step before the first infection (pre-epidemic) and the first time step after the last infected agent has recovered (post-epidemic). It turns out that in a considerable portion of the simulation runs, agents do not see reasons to change their ties given their anticipated benefits and costs of ties even though some of their neighbors are infected by the diseases. By comparing these simulations with the ones in which agents do indeed change ties to distance themselves from others (see I.II. and I.III. in Table 5), we observe that in the runs with network changes attack rate is on average $34.28\%$ lower and duration is on average 2.83 time steps shorter. Furthermore, considering that networks were pairwise stable before the initial infection, the average number of 3.41 network changes per agent (sum of dissolution and creation of ties) is caused and mainly driven by the presence of infectious agents. Additionally, it is shown that even more network changes were initiated by agents trying to connect to others (on average 18.00 tie requests per agent). These tie requests, however, were accepted only in $9\%$ of the cases. This is a vast difference to the network initialization stage prior to the epidemics, where $40\%$ tie requests were accepted from an average of 7.97 tie requests per agent. Despite the many changes in network ties, the overall network measures—with regard to degree at distance 1 and 2, and summary statistics of the density, clustering, and closeness—are the same before and after the epidemics.

**Table 5:** Descriptive statistics of network dynamics, epidemics, and network measures

	Mean	SD	Min	Max	Skew
I. Epidemics					
I.I. All simulation runs (36,000)					
Final size	74.75	36.54	2.00	100.00	$-$1.00
Duration	20.66	6.68	10	60	0.30
I.II. Simulation runs with network changes (23,930)					
Final size	63.26	39.34	2.00	100.00	$-$0.40
Duration	19.71	7.34	10	59	0.43
I.III. Simulation runs without network changes (12,070)					
Final size	97.54	11.57	4.00	100.00	$-$6.49
Duration	22.54	4.58	10	60	1.10
II. Network dynamics					
II.I. During epidemic					
Av. no. ties broken/agent	1.80	2.61	0.00	22.86	2.46
Av. no. ties created/agent	1.81	2.61	0.00	22.80	2.46
Av. no. tie requests/agent	18.95	25.27	0.00	231.82	2.37
% tie requests accepted	0.09	0.03	0.01	1.00	6.21
II.II. Pre-epidemic					
Av. no. tie requests/agent	7.15	6.48	0.00	34.94	1.59
% tie requests accepted	0.41	0.18	0.07	1.00	0.59
III. Network measures					
III.I. Pre-epidemic					
Degree	6.12	2.18	3.00	10.80	0.77
Distance 2 degree	16.88	11.78	4.00	39.68	1.08
Density	0.30	0.07	0.19	0.56	0.17
Clustering	0.01	0.01	0.00	0.27	4.65
Closeness	0.96	0.02	0.93	0.98	$-$0.31
III.I. Post-epidemic					
Degree	6.09	2.11	3.00	10.92	0.75
Distance 2 degree	16.91	11.86	4.00	40.12	1.08
Density	0.30	0.07	0.18	0.56	0.24
Clustering	0.01	0.01	0.00	0.27	4.62
Closeness	0.96	0.02	0.93	0.98	$-$0.31

Tables 6 and 7 show the *Interaction effects* models of Tables 2 and 3 divided by network size. The relatively large effect of degree of patient-0 (${\text{deg}}^{0}_{\text{start}}$) in the model for networks of size 50 in Table 6 (and the accompanying significance reduction of the other parameters) suggests that final size of epidemics in large networks is mostly driven by average degree. The reversal of many main effects between small and large networks for duration (Table 7) shows that in larger and thus denser networks provide more potential transmission routes, ultimately allowing the disease to travel faster. Furthermore, most interaction effects vanish in the $N = 50$ model. Thus, network size drives the entire epidemic dynamics in large networks, while other parameters only have an effect in smaller networks. Regression models of epidemic duration split by network size (Table 6) show that many main effects reverse between small and large networks. Consider, for example, benefits at distance 2 ($\beta$). In the models for networks with $N < 50$ the effect is positive, while in models for networks with $N = 50$ the effect is negative. That is, in large networks increased benefits create even more ties per agent, ultimately providing large numbers of potential transmission routes, and thus allowing the disease to travel faster.

**Table 6:** Comparison of two-level random-intercept logistic regression models for final size of the epidemic by network size

	Combined	N=10	N=15	N=20	N=25	N=50
Fixed effects (observed effects)						
Intercept	2.59*** (0.07)	0.05 (0.04)	1.46*** (0.11)	2.40*** (0.16)	3.17*** (0.19)	4.75*** (0.27)
Main effects						
I. CIDM parameters						
Benefit distance 2 ($\beta$)	0.23*** (0.02)	0.23*** (0.01)	0.25*** (0.03)	0.27*** (0.04)	0.26*** (0.04)	0.20*** (0.04)
Cost increase for infected ties ($\mu$)	− 5.30*** (0.21)	− 4.89*** (0.17)	− 5.85*** (0.42)	− 6.71*** (0.62)	− 6.53*** (0.76)	− 4.44*** (0.94)
Disease severity ($\frac{\sigma }{50}$)	− 1.75*** (0.11)	− 1.86*** (0.10)	− 1.91*** (0.23)	− 2.50*** (0.30)	− 2.11*** (0.31)	− 1.59*** (0.35)
Risk perception (*r*)	− 3.16*** (0.13)	− 2.51*** (0.11)	− 3.16*** (0.26)	− 3.76*** (0.36)	− 4.28*** (0.43)	− 3.57*** (0.51)
Network size ($\frac{N}{50}$)	6.22*** (0.40)					
II. Network properties (start of epidemics)						
Density (${\text{den}}_{\text{start}}$)	− 7.35*** (0.98)	− 4.16** (1.27)	− 11.91*** (2.36)	− 10.00** (3.44)	− 13.64** (5.24)	− 5.72 (20.02)
Degree of patient-0 (${\text{deg}}^{0}_{\text{start}}$)	6.46*** (0.54)	2.73*** (0.45)	5.79*** (0.67)	2.88*** (0.82)	6.40*** (1.07)	17.29*** (3.33)
Interaction effects						
$\beta$ x $\mu$	0.52*** (0.06)	0.46*** (0.05)	0.48*** (0.13)	0.54*** (0.16)	0.50** (0.16)	0.30 (0.16)
$\mu$ x $\frac{\sigma }{50}$	3.16*** (0.44)	1.74*** (0.40)	3.80*** (0.92)	4.70*** (1.18)	3.99** (1.21)	2.39* (1.17)
$\mu$ x *r*	6.41*** (0.49)	4.33*** (0.42)	7.60*** (1.04)	8.34*** (1.44)	9.60*** (1.71)	7.24*** (1.91)
$\frac{\sigma }{50}$ x *r*	− 2.04*** (0.27)	− 3.14*** (0.27)	− 1.93*** (0.57)	− 1.65* (0.69)	− 1.88** (0.73)	− 0.74 (0.71)
*r* x ${\text{den}}_{\text{start}}$	9.39*** (1.52)	− 5.29 (3.03)	13.86* (5.38)	21.26* (8.43)	23.66 (13.10)	32.70 (37.99)
$\frac{N}{50}$ x ${\text{deg}}^{0}_{\text{start}}$	13.79*** (2.30)					
Random effects (unobserved effects)						
$s^{2}$	0.47	0.02	0.44	0.57	0.52	0.25
Log likelihood ($\ell$)	− 11,581.31	− 3092.16	− 2717.52	− 2507.48	− 2277.77	− 926.14
Intraclass correlation ($\rho$)	0.125	0.007	0.118	0.149	0.136	0.072
Observations	36,000	7200	7200	7200	7200	7200
Groups: parameter combinations	360	72	72	72	72	72

*** p < 0.001, **p < 0.01, *p < 0.05, SEs in parentheses

**Table 7:** Comparison of two-level random-intercept linear regression models for duration of epidemics by network size

	Combined	N=10	N=15	N=20	N=25	N=50
Fixed effects (observed effects)						
Intercept	21.23*** (0.19)	18.41*** (0.29)	20.43*** (0.23)	21.48*** (0.18)	22.00*** (0.16)	20.97*** (0.08)
Main effects						
I. CIDM parameters						
Benefit distance 2 ($\beta$)	0.36*** (0.04)	0.59*** (0.10)	0.71*** (0.08)	0.45*** (0.06)	0.19*** (0.05)	− 0.16*** (0.03)
Cost increase for infected ties ($\mu$)	− 3.42*** (0.44)	− 10.68*** (1.15)	− 6.26*** (0.94)	− 2.83*** (0.73)	0.11 (0.65)	2.58*** (0.31)
Disease severity ($\frac{\sigma }{50}$)	− 1.81*** (0.26)	− 3.64*** (0.68)	− 3.08*** (0.56)	− 2.42*** (0.44)	− 1.38*** (0.39)	1.50*** (0.19)
Risk perception (*r*)	− 2.81*** (0.27)	− 5.06*** (0.70)	− 4.35*** (0.57)	− 3.62*** (0.45)	− 2.46*** (0.40)	1.45*** (0.19)
Network size ($\frac{N}{50}$)	− 0.11 (1.15)					
II. Network properties (start of epidemics)						
Density (${\text{den}}_{\text{start}}$)	− 19.93*** (3.20)	− 22.55*** (2.81)	− 33.53*** (4.87)	− 25.41*** (6.60)	− 18.31* (8.19)	− 41.03*** (11.45)
Degree of patient-0 (${\text{deg}}^{0}_{\text{start}}$)	3.31*** (0.62)	2.24* (0.98)	7.35*** (1.38)	1.71 (1.66)	2.57 (1.88)	− 1.67 (2.11)
Interaction effects						
$\beta$ x $\mu$	1.05*** (0.15)	1.71*** (0.38)	2.01*** (0.31)	1.45*** (0.24)	0.56** (0.22)	− 0.47*** (0.10)
$\beta$ x *N*	− 1.06*** (0.13)					
$\mu$ x $den_{start}$	− 38.17*** (5.00)	2.64 (10.54)	− 1.11 (18.65)	30.21 (25.49)	31.33 (31.70)	− 27.52 (45.03)
$\frac{\sigma }{50}$ x *r*	− 4.16*** (0.64)	− 6.74*** (1.67)	− 6.84*** (1.37)	− 6.38*** (1.07)	− 3.98*** (0.95)	3.12*** (0.45)
$\frac{\sigma }{50}$ x *N*	6.48*** (0.93)					
*r* x *N*	8.20*** (0.96)					
$\frac{N}{50}$ x ${\text{den}}_{\text{start}}$	32.97*** (9.35)					
Random effects (unobserved effects)						
$s^{2}$	3.96	5.59	3.57	2.05	1.60	0.30
Log likelihood ($\ell$)	− 113,274.74	− 23,109.20	− 23,355.84	− 23,203.31	− 22,761.55	− 19,558.51
Intraclass correlation ($\rho$)	0.114	0.138	0.087	0.054	0.048	0.022
Observations	36,000	7200	7200	7200	7200	7200
Groups: parameter combinations	360	72	72	72	72	72

*** p < 0.001, **p < 0.01, *p < 0.05, SEs in parentheses

## Abbreviations

CM: Connections Model
HBM: Heath Belief Model
NIDM: Networking during Infectious Diseases Model
SIR (Model): Susceptible, Infected, Recovered (Model)
SPF: Social Production Function

## Footnotes

1 Clearly, the NIDM also allows modeling larger networks. That, however, requires a different social network formation model than the one used in this paper.

2 Note that health behavior is not necessarily ignored in models of disease spread. If, for example, estimation of epidemic parameters, such as $R_{0}$ is based on empirical data, behavior is reflected in the data and thus implicitly included.

3 Note that friendship is merely an illustrative example. The NIDM describes general social network and infectious disease dynamics that can be tied to any context. That is, connections may form and dissolve over time based on health behavior. Thus, if someone gets sick, interactions may discontinue independent of their nature (e.g., going for lunch, having a business meeting, having sexual contact), but have the possibility to recover at a later point in time.

4 For more detailed descriptive statistics, we refer to Table 5 in Appendix.

5 Note that the effect of density should be interpreted in relation to the other network characteristics. One must realize that within the CM-model, density hardly varies given network size and as a result if we analyze data for one network size only and we do not control for the degree of patient-0, there is no effect of density. However, if we control for degree of patient-0 for a given network size, the negative effect can be interpreted as a compensation for the overestimation of the effect of patient-0 for the whole network. If we analyze data for different network sizes simultaneously, the density effect interferes with the effect of network size discussed above.
